# Knowledge and Attitudes on Contraception and Reproductive Health in Women With HIV

**DOI:** 10.1093/ofid/ofae044

**Published:** 2024-01-29

**Authors:** Anna Henricks, Samantha Singal, Dana Hughes, Sean Kelly, Jessica L Castilho, Jamison Norwood

**Affiliations:** Vanderbilt University School of Medicine, Nashville, Tennessee, USA; Vanderbilt University School of Medicine, Nashville, Tennessee, USA; Division of Infectious Diseases, Vanderbilt University Medical Center, Nashville, Tennessee, USA; Division of Infectious Diseases, Vanderbilt University Medical Center, Nashville, Tennessee, USA; Division of Infectious Diseases, Vanderbilt University Medical Center, Nashville, Tennessee, USA; Department of Health Policy, Vanderbilt University Medical Center, Nashville, Tennessee, USA; Division of Infectious Diseases, Vanderbilt University Medical Center, Nashville, Tennessee, USA

**Keywords:** contraception, empowerment, HIV, reproductive health

## Abstract

**Background:**

For reasons not fully explained to date, contraception usage among women with HIV remains low. The aim of our study was to understand attitudes toward and lifetime use of contraception among women with HIV.

**Methods:**

We administered an anonymous, community-informed, voluntary survey to cisgender, English-speaking women with HIV (≥18 years of age) at a Southern urban HIV clinic. It included multiple choice and Likert-scale questions on reproductive health. Participants reported contraception use, recollection of provider conversations about contraception, and perceived empowerment and knowledge regarding reproductive health. We used chi-square and Fisher exact tests to compare attitudes and prior conversations about contraception by age (< vs ≥45 years), race (Black vs non-Black), and lifetime contraception use.

**Results:**

The median age of the 114 participants was 52 years, and 62% of the women identified as Black and 31% as White. Women reported a median of 2 unique family planning methods used throughout life, with oral contraceptive pills being most the common (59%). Only 20% of women reported having ever used long-acting reversible contraception (LARC). Only 56% of women recalled talking with a provider about contraception. Women of non-Black race and those who had used LARC were more likely to remember (72 vs 52%; *P* = .035; 87 vs 56%; *P* = .022; respectively). When asked about preferences, 82% of women age <45 years wanted a nondaily method, and 60% felt uncomfortable with device insertion.

**Conclusions:**

Throughout life, participants reported using a diversity of contraceptives. Only half of women remembered a provider conversation about contraception. Understanding women's preferences regarding contraception should guide counseling.

## Teaser

Women living with HIV use many different contraception methods throughout their life but many do not recall having a discussion of contraception options with their providers. Women living with HIV want contraception that is easily reversed and not taken daily.

Women make up nearly a quarter of adults with HIV in the United States, and a large proportion of these women live in Southern states [1, 2]. In addition to other family planning services, access to reliable contraception is important for women with HIV to improve birth outcomes and reduce maternal mortality. It also has the potential to increase feelings of empowerment over one's reproductive health choices and future, especially at a time when reliable access to reproductive health services remains uncertain [3, 4].

Conversations surrounding contraception among women with HIV have a long and variable history. Historically, in the pre–highly active antiretroviral therapy (ART) era, women with HIV were often encouraged to seek permanent sterilization over other reversible methods of contraception because of provider paternalism and fear of vertical transmission, with some US cohort studies reporting sterilization prevalence among women with HIV at approximately twice the national average [5, 6]. Even in the current treat-all era and despite our knowledge of U = U (undetectable HIV viral load = untransmittable HIV virus), recent studies continue to document a trend of lower prescription contraception use among women with HIV compared with their uninfected peers, as well as a high rate of unintended pregnancies [7, 8, 9]. More specifically, usage rates of long-acting reversible contraception (LARC), which includes intrauterine devices (IUDs) and hormonal implants, among women with HIV remain low in comparison to the national population of women of child-bearing age [10]. Insurance coverage of LARC may also differ by private and public payors and perhaps by region, potentially adding to barriers women with HIV face in accessing those options. Despite this difference in LARC use, attitudes about IUDs and implants are similar between women with and without HIV when surveyed, suggesting an unmet need for effective and accessible contraception in this population [11]. Additionally, single-site studies have shown low rates of documentation of family planning discussions with providers among women with HIV [12]. Which factors are most important in shaping attitudes about contraception choice among women with HIV, how these choices affect women's sense of control over their own reproductive health, and how provider counseling affects contraception use among women with HIV today remain poorly understood. To inform medical providers’ communication regarding contraception, our research aimed to determine attitudes toward contraception options and lifetime use of various contraception methods in women with HIV.

## METHODS

Our study population included cisgender, English-speaking women with HIV who received medical care at the Vanderbilt Comprehensive Care Center (VCCC) in Nashville, Tennessee, between October 2022 and February 2023. In 2022, the VCCC had a total of 3827 patients, 768 (20%) of whom were cisgender women. Of those women, 497 (65%) were ≥45 years of age. The VCCC provides comprehensive primary and HIV specialty care to adults with HIV. Women's health care services at the VCCC include screening for sexually transmitted infections and cervical cancer, mammogram referrals, prenatal care in partnership with Vanderbilt's Maternal Fetal Medicine Department, and family planning services, including administration of injectable contraceptives and IUD insertion. Women were included in the study if they were ≥18 years of age, English-speaking, and volunteered to complete the survey. We excluded women who were non-English-speaking and noncisgender. Participants were approached during their clinic visits to take a survey and could complete the survey on an electronic tablet or have the questions read to them by a study investigator. Total time to complete the survey was ∼10 minutes.

The survey included multiple choice and Likert-style questions addressing topics in reproductive health, including methods of family planning. As published surveys of reproductive health attitudes and experiences among women with HIV remain sparse, our survey was developed by study investigators following review of research literature. It was further refined through a community-engaged research workshop where a panel of women with HIV living in the Nashville area piloted the survey and provided input on existing and missing topics and questions as well as language, length, and content. Through survey questions, participants reported past and current use of contraception, satisfaction with each method, preferences about contraception characteristics, and whether they had spoken with either their primary care or HIV provider about contraception. Women were also asked to rate their level of agreement with the following statements: “I feel informed about my reproductive health” and “I feel empowered to make choices about my reproductive health options.”

In this analysis, we first sought to evaluate lifetime contraception use among participants. We calculated the frequency of lifetime use for each type of contraception, including both current and past use of each method. Contraception forms assessed in the survey included bilateral tubal ligations, hysterectomies, oral contraceptive pills (OCPs), IUDs, hormonal implants, injections (including depot medroxyprogesterone acetate), hormonal vaginal rings, hormonal patches, male and female condoms, cervical caps, cervical diaphragms and sponges, natural methods, chemical methods, withdrawal, plan B pills, and no contraception. Contraception forms were also grouped into World Health Organization (WHO) tiers, which range from Tier 1 to Tier 3, with Tier 3 being the least effective at preventing pregnancy and Tier 1 being the most effective [13]. Tier 1 includes IUDs, hormonal implants, tubal ligations, and hysterectomy. Tier 2 includes OCPs, injectable hormones, vaginal rings, and hormonal patches. Tier 3 includes male and female condoms, withdrawal, natural methods, chemical methods, cervical caps, diaphragms, sponges, and plan B. We stratified women into those who had ever used a method from each WHO tier. Given the differences in reversibility, we separated Tier 1 into LARC and the surgical interventions of tubal ligation and hysterectomy. Next, we calculated the frequency of participants who reported speaking with a medical provider about their contraception options, with the response options being “yes,” “no,” or “I don’t remember.” The survey clarified that “medical provider” could refer to a primary care clinician, OBGYN, or HIV provider. We assessed the likelihood of reporting prior provider conversations by various demographic and clinical factors, including age (stratified into < vs ≥45 years), race (stratified to Black vs non-Black), and lifetime contraception use with chi-square tests. Additionally, we analyzed the frequency of provider conversation about specific contraception types. Lastly, we focused on women's overall feelings about reproductive health and personal preferences related to contraception choice. We examined women's preferences using a Likert scale that assessed agreement (strongly disagree, disagree, neutral, agree, strongly agree) with feeling informed and empowered about their reproductive health by demographic and clinical factors using the Fisher exact tests. For women under the age of 45 only, we ranked preferences about contraception by proportion of participants who strongly agreed or agreed with a list of statements related to various contraception characteristics. All analyses were conducted using Stata, version 17.0, Basic Edition. All *P* values were 2-sided.

### Patient Consent

As survey responses were anonymous and participation was voluntary, patient written consent was not required. This study and survey were approved by the Vanderbilt University Institutional Review Board and conform to standards currently applied in the United States.

## RESULTS

A total of 114 women completed the survey. Overall demographic and clinical characteristics are shown in Table 1. The median age was 52 years, and the median age at HIV diagnosis was 31 years. More than half of all participants identified as Black, followed by almost one-third who identified as White. Nearly all participants were receiving ART at the time of the survey. When asked if a provider had ever discussed the concept of U = U with them, a quarter were unsure or did not recall. The median number of lifetime pregnancies was 3. Of all women, almost half were single, and one-third were married. In terms of sexual activity, 38% of respondents indicated that they were currently sexually active. Of participants under 45 years of age, 33% indicated they would be “very unhappy and disappointed” if they were to become pregnant today.

**Table 1.** Demographic and Clinical Characteristics of Survey Participants (n = 114)

	Total Respondents	Median/No.	IQR/%
Age, y	114	52	43, 60
Race/ethnicity	114	…	…
White	…	35	30.7
Black	…	71	62.3
Latina (any race)	…	5	4.4
Asian	…	1	0.9
Prefer not to say	…	1	0.9
Other or >1 race	…	1	0.9
Age at HIV diagnosis, y	107	31	25, 40
Currently receiving ART	114	111	97.4
Lifetime number of pregnancies	113	3	2, 4
Currently pregnant	114	6	5.4
Currently sexually active	114	43	37.7
Relationship status^a^	114	…	…
Single	…	52	45.6
Not in a relationship	…	4	3.5
Dating	…	6	5.3
Married	…	34	29.8
In a committed relationship	…	10	8.8
Widowed	…	4	3.5
Other	…	4	3.5
Provider ever explained U = U?	112	…	…
Yes	…	83	74.1
No	…	20	17.9
Unsure	…	9	8.0

Abbreviations: ART, antiretroviral therapy; IQR, interquartile range; U = U, undetectable HIV viral load equals untransmittable HIV virus.

^a^Women could select all relationship status options that applied to them.

**Figure 1.**
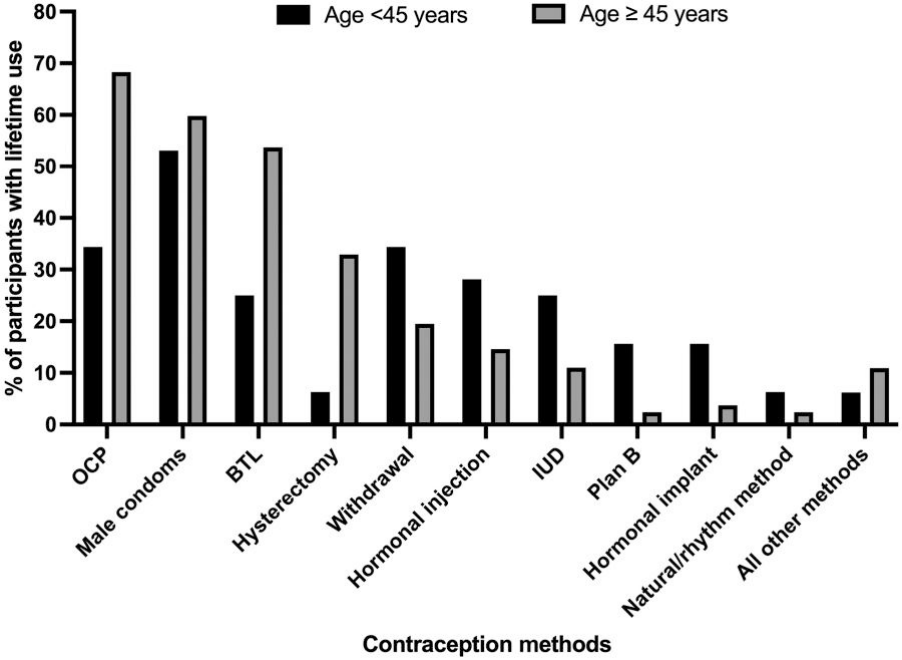
Frequency of lifetime contraceptive use by type among survey participants by age (n = 114). Total percentages exceed 100% because women were able to select multiple options to reflect all contraception methods used throughout their lifetime. “All other methods” includes vaginal rings (n = 1, 0.9%), chemical methods (n = 1, 0.9%), hormonal patches (n = 0), and cervical caps, diaphragms, and sponges (n = 1, 0.9%). Abbreviations: BTL, bilateral tubal ligation; IUD, intrauterine device; OCP, oral contraception pills.

Women reported a median (interquartile range) of 2 (1–3) unique family planning methods used during their lifetime. Overall, OCPs were the most frequently reported type of contraception ever used by all women, followed by tubal ligation, hysterectomy, and withdrawal (Figure 1). Looking specifically at women under 45 years of age, male condoms were most common (53%), followed by OCPs (34%) and withdrawal (34%). In total, 56% of all respondents had used a form of permanent sterilization. Of those under 45 years of age, 6% had undergone a hysterectomy and 25% had undergone a tubal ligation, compared with 33% and 54% of women over 45 years of age. The median age at procedure among all women was 37 years for hysterectomy and 27 years for tubal ligation. Of women who reported both age at procedure and age at HIV diagnosis, half of women underwent a tubal ligation after their HIV diagnosis (25/50 women), while two-thirds of women underwent their hysterectomy after HIV diagnosis (18/27 women). Approximately 20% of women had utilized a LARC at any point, with highest lifetime use among women <45 years of age.

When stratified by WHO tier, few women had only used a method from 1 single tier during their life, with 6% only using a WHO Tier 1 method during their lifetime, 6% only using a WHO Tier 2 method, and 11% only using a WHO Tier 3 method. Examining whether participants had ever used a method from each tier, 69% of women overall had used a Tier 1 method, 67% had used a Tier 2 method, and 64% had used a Tier 3 method. Most women had used methods from >1 tier throughout their lifetime, with 45% using both Tier 1 and Tier 2, 46% using both Tier 1 and Tier 3, 43% using both Tier 2 and Tier 3, and 30% using a method from all 3 tiers.

Among all women, 56% recalled talking with a medical provider about contraceptive options, and 44% did not or were unsure. Among women who recalled a conversation, OCPs (recalled in 41/60 women or 68%) and male condoms (recalled in 27/60 women or 45%) were the most discussed methods. Hormonal vaginal rings (recalled in 2/60 women or 3%), withdrawal (recalled in 2/60 women or 3%), and female condoms (recalled in 2/60 women or 3%) were the least discussed contraception options. For only women under 45 years of age, IUDs were the most discussed method. Table 2 shows the factors associated with recalling a conversation about contraception with their medical provider. Women of non-Black race and those who had used a LARC were statistically more likely to remember having this conversation. A greater proportion of younger women remembered family planning conversations with providers: 69% of women under 45 years of age vs 56% of women over 45 years of age. However, neither age nor prior use of specific WHO tier contraception was statistically associated with recollection of a provider conversation about contraception.

**Table 2.** Characteristics of Participants who Reported “Yes” or “No or Unsure” to Past Conversation With Provider About Contraception Options (n = 114)

	Yes	No or Unsure	*P* Value^a^
Age, No. (%)	…	…	.22
<45 y	22 (68.8)	10 (31.3)	…
≥45 y	46 (56.1)	36 (43.9)	…
Race, No. (%)	…	…	.035
Black	37 (52.1)	34 (47.9)	…
Non-Black	31 (72.1)	12 (27.9)	…
Lifetime Tier 1 use,^b^ No. (%)	…	…	.72
Yes	48 (60.8)	31 (39.2)	…
No	20 (57.1)	15 (42.9)	…
Lifetime Tier 2 use,^c^ No. (%)	…	…	.28
Yes	48 (63.2)	28 (36.8)	…
No	20 (52.6)	18 (47.4)	…
Lifetime Tier 3 use,^d^ No. (%)	…	…	.86
Yes	44 (60.3)	29 (39.7)	…
No	24 (58.5)	17 (41.5)	…
Lifetime LARC use,^e^ No. (%)	…	…	.022
Yes	13 (86.7)	2 (13.3)	…
No	55 (80.9)	44 (95.7)	…

Abbreviation: LARC, long-acting reversible contraception.

^a^Chi-square test of comparison of proportions.

^b^Lifetime use of any of the following forms of contraception: intrauterine device, hormonal implants, tubal ligations, and hysterectomy.

^c^Lifetime use of any of the following forms of contraception: oral contraceptive pills, hormonal injections (including depot medroxyprogesterone acetate), hormonal vaginal rings, hormonal patches.

^d^Lifetime use of any of the following forms of contraception: male and female condoms, cervical caps, cervical diaphragms and sponges, natural methods, chemical methods, withdrawal, plan B pills.

^e^Lifetime use of any of the following forms of contraception: intrauterine device and hormonal implants.

Women were asked about their contraceptive preferences by reporting agreement (“agree” or “strongly agree”) with the importance of various contraceptive characteristics. Results of preferences for women under the age of 45 are shown in Table 3. Overall, most women under 45 years of age preferred a contraception method that is not taken daily, that is easily stopped or reversed, that they “don’t have to think about,” and that reduces the flow and frequency of their menstrual cycle. Over half felt uncomfortable with insertion of a medical device for contraception. Few women were worried about their contraception interacting with their HIV medications or were concerned about future fertility or the cost of contraception.

**Table 3.** Preferences Related to Choosing a Contraception Option Among Participants <45 Years of age (n = 17)

How Strongly Do You Agree or Disagree With the Following Statements About Your Current Birth Control Options?	Total No. of Respondents	Strongly Agree or Agree, No. (%)
I don’t want to take it every day	17	14 (82)
I want it to be easily stopped or reversed	16	12 (75)
I don’t want to have to think about it	16	12 (75)
I prefer a method that reduces the flow and frequency of my menstrual cycle	16	11 (69)
I am uncomfortable with a medical device being inserted	15	9 (60)
I want a method that is comfortable for my partner	15	9 (60)
I don’t want a method that interrupts sexual activity	17	8 (47)
I am worried about possible side effects	17	8 (47)
I don’t have sex often enough to use daily	14	5 (36)
I am worried it will interact with my HIV medications	15	3 (20)
I prefer a method that allows me to have a period every month	16	3 (19)
I am worried about effects on my sex drive or satisfaction	14	2 (14)
I am worried it will affect my chances of becoming pregnant in the future	15	1 (7)
I am worried about how much it costs	15	1 (7)

Lastly, the majority of women participating in this survey strongly agreed or agreed with feeling empowered (88%) and informed (83%) about their reproductive health. Compared with women ≥45 years of age, those <45 years felt more informed (81.01% vs 87.50%; *P* = .045). There were no statistically significant associations between race, lifetime contraception use by WHO tier, or lifetime LARC use and feelings of empowerment or being informed regarding reproductive health (*P* > .05 for all, data not shown).

## DISCUSSION

We observed striking trends in this community-informed survey study of contraception use and preferences among women with HIV and of care in the US South. Overall, and consistent with other studies, women in our clinic reported a diversity of use of prior contraception methods, though only a minority reported use of long-acting reversible forms or injectable hormones. Many women did not recall having conversations with their providers about contraception. Though many women desired contraceptive options that were nondaily and reversible, many were also uncomfortable with implantable medical devices. Our findings underscore the importance of ongoing and patient-centered discussions about contraception between women with HIV and their medical providers.

Throughout their lives, women with HIV who participated in this study used a diversity of contraceptive methods. A previous longitudinal study of >2500 women with HIV found that barrier methods of contraception were most common, followed by sterilization, with <10% of women using a form of hormonal contraception [14]. In contrast, OCPs were the most commonly used method overall in our study, followed closely by barrier methods and then permanent sterilization. One reason for this may be that women did not select condom use in our survey even if they had previously used this method because they may not have considered condoms to be for prevention of pregnancy. When examining contraception use by WHO tiers, relatively few women reported that they had only used a method from a single tier during their lifetime, and almost a third of respondents had used a method from all 3 tiers. Women with HIV likely use multiple different contraception methods during their lifetimes, and thus repeated counseling about contraception options would be valuable. However, when asked in the study about prior conversations with their providers about contraception options, almost half of women surveyed did not remember a conversation. Of women <45 years of age, one-third did not remember a conversation. Our results suggest that the reproductive counseling needs of women with HIV may not be currently met. This finding is consistent with other studies, including 1 study among women with HIV that demonstrated that 56% of women wanted reproductive counseling from their provider but had not received it [15]. In our study, when women did remember a provider conversation, they most often remembered being presented with condoms and OCPs as contraception options. This indicates that not only should counseling be happening more often, but that it should also include a discussion of the full range of methods available that best match patients’ desired characteristics for contraception.

When asked about contraception preferences in our study, most women preferred a nondaily method but felt uncomfortable with insertion of a device. Additionally, they desired a method that is easily reversible. While an IUD or hormonal implant can be effective for years to prevent pregnancy and can be removed easily, both methods do require an in-office procedure for insertion. Additionally, 58% of women under 45 years of age who recalled a provider conversation remembered the provider discussing an IUD, but preferences in this group indicate that there is still a sense of discomfort with device insertion for contraception. Injectable hormonal contraception offers a nondaily option, but use in our cohort remained relatively low (15% among women <45 years of age). The vaginal ring or hormonal patch also requires only weekly exchanges, which can be done by the patient at home. However, the majority of participants in our study did not remember a provider ever discussing either of these options. Interestingly, one study at a public health clinic demonstrated that introduction of provider training on contraception counseling resulted in more patients being interested in LARC and a substantial increase in the number of patients discussing contraception with their doctor [16].

While some small studies have demonstrated interactions between certain antiretroviral therapies and oral contraceptive pills or hormonal injections, the most recent guidelines from the World Health Organization indicate no absolute contraindications to any form of contraception based on HIV status alone [17, 18, 19]. Overall, patient preferences and shared decision-making principles should guide future counseling. Additionally, patient attitudes toward certain contraception methods, specifically long-acting or implantable methods, may help guide the development and implementation of long-acting ART.

Reassuringly, most women participating in our study reported feeling empowered and informed regarding their reproductive health. Several studies in African countries have shown a relationship between contraception use and feelings of sexual empowerment, but there is limited information about this same relationship in US women or women with HIV specifically [20, 21]. The high prevalence of empowerment and feeling informed among women in our study may be due to their connection to comprehensive HIV and primary care or may reflect a selection bias among the women who chose to volunteer to participate in a reproductive health survey.

While our study offers novel and important insights into contraception use and preferences among women with HIV, there are some important limitations to consider. First, our survey was entirely voluntary and may have been subject to selection bias among those who chose to participate. It is possible that the perspectives of women who may feel less comfortable discussing their reproductive health were not included. The attitudes of women with HIV who are not engaged in longitudinal HIV care and those who were non-English-speaking were also not included. Further, persons assigned female at birth but who do not identify as cisgender women were not included. Future studies should attempt to focus on reaching populations who may have less access to reproductive health care and those from a diversity of cultural and language backgrounds. We did not gather data about the total number of women approached for the survey, so were unable to calculate a survey response rate. To maintain anonymity for participants when discussing sensitive topics related to reproduction and sexuality, we did not collect identifying information in order to conduct medical record review to determine timing of contraception use or sterilization in relation to HIV diagnosis. Future studies are needed to determine how HIV diagnosis may affect women's choice of family planning options, as well as which specific barriers women with HIV face to access of certain forms of contraception. Similarly, we also were not able to address the specific reasons for usage of each form of contraception, such as methods that may be used for both contraception and sexually transmitted infection prevention. Additionally, consistent with our clinic population, participants in our study had a median age of 52 years and may not have had conversations about contraception or used contraception in many years. Their responses may have also been subject to recall bias given this duration. To address this limitation, we focused on women <45 years of age when able, albeit we were limited by a small total population. Lastly, the results of our survey reflect only the clinic population and the VCCC and may not be generalizable to other cities and settings. Further studies on this topic are needed from a diversity of clinical settings serving women with HIV.

In conclusion, our results indicate that more frequent and patient-centered counseling on contraceptive options for women with HIV is needed to enshrine adequate access to reproductive health services for this population. Provider and patient education about various contraception options is also needed to facilitate these conversations and ensure that women with HIV can make informed decisions about their reproductive health.

## Notes

***Acknowledgments.*** First, we thank the women who participated in our study and those who shared their powerful voices and perspectives with us. We acknowledge the faculty and staff at the Vanderbilt Comprehensive Care Clinic for their support of this project and its implementation. We also recognize the contributions of the Vanderbilt Institute for Clinical and Translational Research for their organization of our community-engaged research studio and the time and candor of the women with HIV who participated in the workshop. We acknowledge Dr. Elizabeth Rose at Vanderbilt University Institute for Global Health for her guidance and advice throughout this project. We appreciate the support and research guidance provided by research nurse Morgan Lima, RN, of the Clinical Core of the Tennessee Center for AIDS Research.

***Financial support*.** This work was supported by the Vanderbilt University School of Medicine and the National Institutes of Health (the Tennessee Center for AIDS Research (P30 AI110527) and the Vanderbilt Institute for Clinical and Translational Research (UL1 TR000445)).
